# GPU-accelerated iterative reconstruction for limited-data tomography in CBCT systems

**DOI:** 10.1186/s12859-018-2169-3

**Published:** 2018-05-15

**Authors:** Claudia de Molina, Estefania Serrano, Javier Garcia-Blas, Jesus Carretero, Manuel Desco, Monica Abella

**Affiliations:** 1 Departamento de Bioingeniería e Ingeniería Aeroespacial, Universidad Carlos III de Madrid, Madrid, Spain; 2 Instituto de Investigación Sanitaria Gregorio Marañón (IiSGM), Madrid, Spain; 3 Computer Architecture and Technology Area, Universidad Carlos III de Madrid, Madrid, Spain; 4 Centro de Investigación Biomédica en Red de Salud Mental (CIBERSAM), Madrid, Spain; 5 Centro Nacional Investigaciones Cardiovasculares Carlos III (CNIC), Madrid, Spain

**Keywords:** GPU, Memory management, Parallel processing, Iterative reconstruction, Split Bregman, Limited-data tomography, CBCT

## Abstract

**Background:**

Standard cone-beam computed tomography (CBCT) involves the acquisition of at least 360 projections rotating through 360 degrees. Nevertheless, there are cases in which only a few projections can be taken in a limited angular span, such as during surgery, where rotation of the source-detector pair is limited to less than 180 degrees. Reconstruction of limited data with the conventional method proposed by Feldkamp, Davis and Kress (FDK) results in severe artifacts. Iterative methods may compensate for the lack of data by including additional prior information, although they imply a high computational burden and memory consumption.

**Results:**

We present an accelerated implementation of an iterative method for CBCT following the Split Bregman formulation, which reduces computational time through GPU-accelerated kernels. The implementation enables the reconstruction of large volumes (>1024^3^ pixels) using partitioning strategies in forward- and back-projection operations. We evaluated the algorithm on small-animal data for different scenarios with different numbers of projections, angular span, and projection size. Reconstruction time varied linearly with the number of projections and quadratically with projection size but remained almost unchanged with angular span. Forward- and back-projection operations represent 60% of the total computational burden.

**Conclusion:**

Efficient implementation using parallel processing and large-memory management strategies together with GPU kernels enables the use of advanced reconstruction approaches which are needed in limited-data scenarios. Our GPU implementation showed a significant time reduction (up to 48 ×) compared to a CPU-only implementation, resulting in a total reconstruction time from several hours to few minutes.

## Background

The source-detector pair in conventional cone beam computed tomography (CBCT) systems rotates around the patient through 360 degrees (full angular span) to acquire at least 360 projections. However, there are cases in which the number of projections acquired is smaller and/or covers a smaller angular span (down to 150 degrees) owing to movement limitations, as occurs during surgery, or in respiratory-gated CT, where only a few projections correspond to each gate. The reconstruction of these limited data with the conventional method proposed by Feldkamp, Davis and Kress (FDK) results in severe artifacts in the image (streaks and/or edge distortion), making it advisable to use advanced reconstruction methods that compensate for the lack of data by including prior information about the sample. The most common option for prior information is the assumption of local smoothness, which can be imposed by adding the minimization of the L_1_ norm of the total variation (TV) term. Since the TV term is not differentiable, the use of traditional reconstruction methods may be subject to instability problems [1]. In [2], the authors showed that reconstructing limited data in CT can be efficiently solved by means of the Split Bregman formulation, which reduces the optimization problem to a sequence of unconstrained and simpler problems that are updated iteratively.

In a previous work [3], we presented a new reconstruction method based on the Split Bregman formulation. We reported significant image improvement in terms of artifact reduction using this approach for limited-data CBCT, as compared with FDK. We have presented two implementations of this algorithm, one combining MATLAB and CUDA [3] and another one based on a CPU distributed version [4]. The main limitation of both solutions is that only 2D images can be reconstructed owing to computational and memory requirements. Reconstruction of 3D images with these methods was not possible for two main reasons: (1) memory requirements of the algorithm, and (2) long execution times which hinder the reconstruction of standard size volumes in a reasonable amount of time.

Another example of using MATLAB and CUDA is the work by Smith et al. [5], an iterative reconstruction method based on Split Bregman for MRI. The main limitation of this work is that the communication between MATLAB and GPUs is done through an intermediate library, which increases the overhead with respect to programming in native languages. Furthermore, MRI reconstruction uses FFT (Fast Fourier Transform), which is computationally less expensive than the projection and backprojection kernels needed in CT reconstruction.

Other works presented CPU-GPU implementations using native languages for FDK [6–8], a reconstruction method less challenging than iterative reconstruction. Regarding iterative reconstruction algorithms, which include several projection and backprojection operations, techniques employed for parallelization highly affect the reconstruction execution time as shown in [9], obtaining a speedup factor between 50 × and 200 × using two GPUs with respect to the execution of the same algorithm in a single-thread CPU. Hu et al. [10] proposed an advanced multi-resolution approach to reduce the total execution time. Nevertheless, this work was applied to full span data with a high number of projections. Focusing on the problem of limited data, Jia et al. [11] proposed a new iterative method but they did not address the problem of handling large volumes. A more recent work by Matenine et al. [12] presented a solution for reduced number of projections, but the authors commented the limitation by the memory capacity of the GPUs. Nevertheless, none of these works addressed the problem of limited angular span.

In this work, we present an accelerated implementation for limited data both in angular span and number of projections, that uses the GPU for the most time-consuming operations. Our solution includes a partitioning strategy to be able to handle large volumes with a total footprint of several GB.

## Implementation

### Algorithm

The reconstruction problem follows the TV minimization [13]:

1 $$  min\left \| \nabla (u)\right \|_{1} \quad\! s.t.\left \| Au - f \right \|_{2}^{2} \leq \sigma^{2},\quad\! u \geq 0,\quad\! u \in \Omega  $$

where ∥∇(*u*)∥_1_ corresponds to the *L*_1_ norm of the gradient of the reconstructed image *u*, *A* is the system matrix, *f* is the acquisition data, *σ*^2^ is the image noise, and *Ω* is the subspace corresponding to the field of view (FOV).

Using the Split Bregman formulation [1], the *L*_1_-constrained optimization problem shown in Eq. (1) can be converted into the following unconstrained problems, which are solved at each iteration *k*:

2 $$  {{\begin{aligned} \left(u^{k+1}, d_{x}^{k+1}, d_{y}^{k+1}\right) = min\left \| (d_{x}, d_{y}) \right \|_{1} + \frac{\mu}{2} \left \| Au -f^{k} \right \|_{2}^{2} + \\ +\frac{\lambda}{2} \left \| d_{x} - \nabla_{x} u - b_{x}^{k} \right \|_{2}^{2} + \frac{\lambda}{2} \left \| d_{y} - \nabla_{y} u - b_{y}^{k} \right \|_{2}^{2} \end{aligned}}}  $$

3 $$ f^{k+1} = f^{k} + f - Au^{k+1}  $$

4 $$ b_{x}^{k+1} = b_{x}^{k} + \nabla_{x}u^{k+1} - d_{x}^{k+1}  $$

5 $$ b_{y}^{k+1} = b_{y}^{k} + \nabla_{y}u^{k+1} - d_{y}^{k+1}  $$

where *μ* and *λ* are regularization parameters. Equation (2) can be split into two sub-problems. The first sub-problem contains only differentiable *L*_2_-norm terms. By differentiating with respect to *u* and setting the result to 0, we obtain the following problem:

6 $$  \left(\mu A^{T} A-\lambda \nabla^{T} \nabla \right) u^{k+1}=\mu A^{T} f^{k}+\lambda \nabla^{T} \left(d^{k}-b^{k}\right)  $$

which can be summarized in the following problem:

7 $$ Ku^{k+1} = rhs^{k}  $$

which is solved iteratively using a Krylov space solver, namely, the biconjugate gradient stabilized method. In this step, an input parameter *β* controls the stability of the problem. The second sub-problem contains *L*_1_ terms that are not differentiable. Therefore, it is tackled using analytical formulas (shrinkage operation), which need two additional input parameters *α* and *λ*. Finally, Eqs. (3, 4, 5) are the Bregman iterations that impose constraints for acquired data and total variation, respectively.

### Parallel implementation

**Fig. 1:**
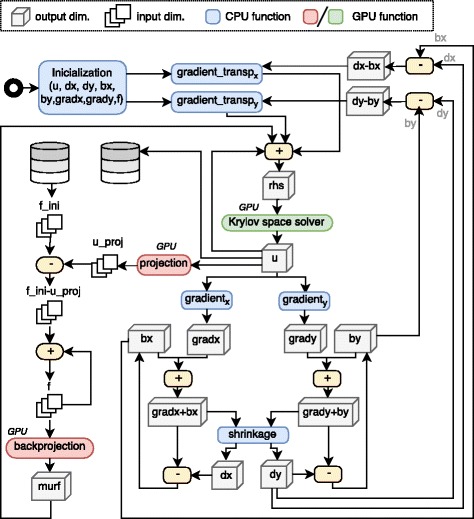
TV3D iterative reconstruction workflow

**Fig. 2:**
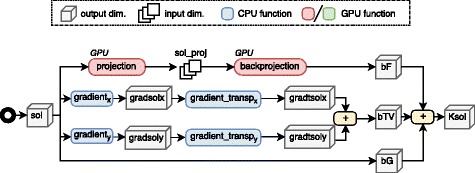
Workflow of the callback function for Krylov solver

The accelerated implementation proposed (written in C and CUDA) is described in Algorithms 1 and 2 and its workflow representation shown in Figs. 1 and 2, respectively. These algorithms are executed iteratively in two nested loops.

The system matrix *A* and its transpose are substituted by a ray-driven projector and a voxel-driven backprojector, which are applied at each iteration a variable number of times, depending on the convergence of the Krylov space solver. Given that those algorithms represent the main computational burden of the method, we implemented them as accelerated kernels that run on GPUs. Other operations that run on GPU are the gradients (*gradient*_*x*_, *gradient*_*y*_), the shrinkage operation (*shrinkage*), and the *L*_2_-norm calculation (using CUBLAS library). The remaining element-wise operations are vectorized by the compiler [14] and multi-thread CPU parallelized with OpenMP 4.0.





The division of the main problem into simpler sub-problems from the Split Bregman formulation results in the need for allocating up to eight times the memory corresponding to the desired output volume, resulting in a total memory footprint of several GB. Given GPU memory restrictions, we implemented a partitioning strategy in both backprojection and projection operations, which are the ones that require the highest amount of memory. With this strategy, input and output data are divided into chunks, and the memory is allocated dynamically.

The Krylov space solver is implemented with the biconjugate gradient stabilized method, BiCGStab [15], where the input matrix in Eqs. (6, 7) is substituted by the Algorithm 2.

## Results

**Fig. 3:**
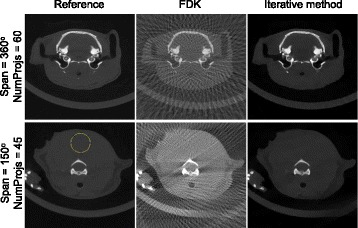
From left to right: reference image and reconstructed image with the FDK-method and the proposed iterative method. Top panel corresponds to the case of 60 projections covering an angular span of 360 degrees and bottom panel to the case of 45 projections covering an angular span of 150 degrees. Yellow circle in the bottom left panel shows the ROI for the SNR measurement

The method was evaluated in a computer with two Intel(R) Xeon(R) E5-2630 v3 processors at 2.40 GHz and one NVidia Tesla K40c GPU. Limited-data acquisitions (*DimProj*×*DimProj*×*NumProjs* pixels) were simulated from a previously acquired small-animal scan (512×512×512 pixels; 0.125 mm pixel size), as shown in Fig. 3, left. We studied the following parameters: dependency on the number of projections with *NumProj* = 45, 60, 90, and 120 covering an angular span of 360 degrees and *DimProj*=512; dependency on angular span for *NumProj*=45 uniformly distributed in an angular span of 45, 60, 90, 135, 150, 180, and 270 degrees (*DimProj*=512); and the effects of the projection size, by considering *DimProj* = 256, 512, and 1024 when 90 projections are obtained uniformly distributed in an angular span of 360 degrees. All simulations were generated using FUX-SIM [16], a simulation/reconstruction framework for X-ray systems.

These data were reconstructed with an FDK-based method [17] and the proposed iterative method resulting in a volume of *DimProj*×*DimProj*×*DimProj* pixels. For the latter, we used *α*=0.003, *μ*=20, *β*=3, and *λ*=2 as reconstruction parameters (see [2] for details on how to select these parameters). The number of iterations (*iterations* in Algorithm 1, line 4) was 35, selected high enough to ensure an error variation smaller than 1%.

**Fig. 4:**
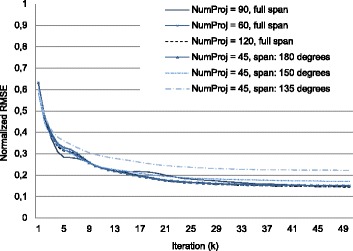
RMSE vs. iterations for 60, 90 and 120 projections (full span) angular span of 135, 150 and 180 degrees (45 projections)

Figure 3 shows the reference image (FDK reconstruction of the complete dataset) and the results of FDK and the proposed iterative method for two limited-data configurations. Image quality was assessed with two metrics. To evaluate the global image quality, we calculated the root mean square error (RMSE) between the reference image and the intermediate solution *u*^*k*^ from the limited dataset. To evaluate the influence of streaks and noise in the reconstructed image, we measured the improvement of signal to noise ratio (SNR) obtained with the iterative method with respect to the FDK-based method in the homogeneous area indicated in Fig. 3. Table 1 shows both metrics for different number of projections and angular span, with a noticeable improvement when increasing the angular span despite the low number of projections. Figure 4 plots the dependence of the RMSE with the number of iterations, *k*, for six different limited-data cases varying the angular span and the number of projections. We can see that the proposed iterative method shows a similar behaviour for all limited-data configurations.

**Table 1:** SNR difference in dB between the FDK and the iterative reconstruction; RMSE between the iterative reconstruction and the reference image for different limited-data configurations

Angular span	Projections	SNR Difference (dB)	RMSE
135	45	20.79	0.268
150	45	23.20	0.220
360	45	28.27	0.154
360	90	26.17	0.153
360	120	25.98	0.151

**Fig. 5:**
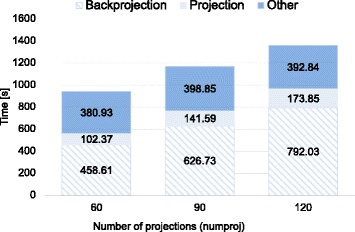
Execution time (in seconds) for different number of projections (*NumProj*)

**Fig. 6:**
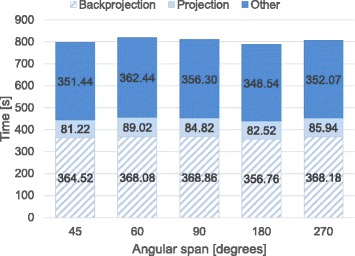
Execution time (in seconds) for different angular span (degrees)

**Fig. 7:**
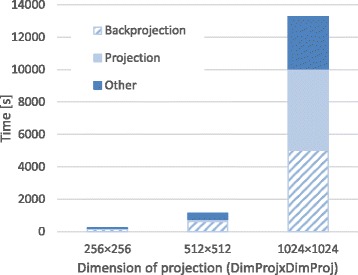
Execution time (in seconds) for different projection size (*DimProj*)

Figures 5, 6, and 7 show the breakdown of the reconstruction time of each configuration, obtained as the average of three consecutive executions in order to avoid time variability due to operating system operations. Reconstruction time is divided into backprojection, forward projection, and time spent in other operations including I/O operations and CPU computation.

**Fig. 8:**
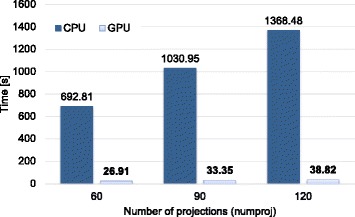
Execution time (in seconds) of the first iteration for both CPU and GPU implementations for different number of projections (*NumProj*)

**Fig. 9:**
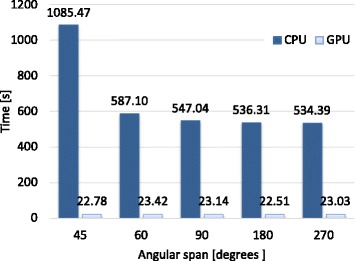
Execution time (in seconds) of the first iteration for both CPU and GPU implementations for different angular span (degrees)

Finally, we compared our implementation in GPU of the iterative method with a CPU-only implementation of the same iterative method parallelized using OpenMP to fully exploit multi-core architectures. Figures 8 and 9 plot the time spent in the first iteration (average of three different executions) reaching a speedup factor of 48 × with the GPU implementation with respect with the CPU-only one.

## Discussion and conclusions

We present an accelerated implementation of a method for 3D limited-data tomography solved in an efficient way by using a GPU for the most time-consuming operations.

Our evaluation of the method showed a high reduction of the severe artifacts present when using the conventional FDK-based method for cases with low number of projections, with an SNR improvement better than 20 dB for all cases. The image distortion due to the limited angular span was also reduced with the proposed method.

To evaluate the performance of the implementation according to data size, we fixed a high number of iterations (*iterations*=35) for all experiments in order to ensure optimum image quality for the worst conditions. Nevertheless, in some cases, the number of iterations could be lowered, resulting in shorter reconstruction times: for example, with 60 projections and an angular span of 360 degrees, 20 iterations were enough for a high quality image. In all experiments, backprojection and forward projection operations represented at least 50% of total execution time, reaching a maximum of 80% of the time when the acquired data set is large. Neither the iterative Krylov space solver nor reading and writing operations significantly increase total time. The execution time of the proposed implementation varies linearly with the number of projections and does not depend significantly on the angular span. The size of the input projections results in a quadratic increase in total computing time.

Reconstructions of large studies (volume of 1024×1024×1024 pixels) are feasible with this accelerated implementation of the iterative method thanks to the partitioning strategy followed for both backprojection and forward projection operations.

Our GPU implementation showed significant time reduction (up to 48 ×) compared with a CPU-only implementation, resulting in a decrease of the total reconstruction time from several hours to few minutes. A fair comparison with other iterative reconstruction implementations proposed in the literature is not feasible owing to differences in the specific algorithms and the hardware used. Nevertheless, we note that the work by Matenine et al. [12], which is the most similar to our solution, was limited by the memory capacity of the GPUs and did not address the problem of limited angular span. In contrast, our GPU accelerated algorithm obtains similar results in terms of execution time despite the fact that it works with large detector and reconstructed volume sizes with a low number of projections in a limited angular span, which increase significantly the number of iterations needed for convergence.

Regarding our previous implementations of the same algorithm, the implementation we propose substantially reduces reconstruction time and hardware resources. As previously reported [3], a solution combining MATLAB and CUDA kernels required a large amount of memory transfers between the CPU and the GPU, resulting in increased execution times, which is unfeasible for the large 3D volumes in real scenarios. This problem is partially solved here due to the use of native code and explicit memory transfers. On the other hand, the complete CPU-based implementation presented in [4] required a high volume of distributed resources to obtain acceptable execution times. For example, using 12 compute nodes resulted in more than 1,000 seconds for a volume of 512×512×512 pixels for only 2 iterations of the algorithm, which is insufficient to obtain a high-quality image.

Efficient implementation using parallel processing and large-memory management strategies together with GPU kernels enables the use of advanced reconstruction approaches which are needed in limited-data scenarios.

## Availability and requirements

**Project name:** RecoItTV**Project home page:**https://github.com/arcosuc3m/recoittv**Operating Systems(s):** Windows, Linux, MacOS**Programming language:** C**Other requirements:** NVidia CUDA must be installed.**License:** Creative commons Non Commercial
